# Exploring Eco-Friendly Microbial Strategies for Nosemosis Control in Honeybee

**DOI:** 10.3390/microorganisms13102357

**Published:** 2025-10-14

**Authors:** Bogdan Tache, Roxana Spulber, Laura-Dorina Dinu, Emanuel Vamanu

**Affiliations:** 1 Research and Development Institute for Apiculture, 013975 Bucharest, Romania; bogdan.tache@icdapicultura.ro (B.T.); roxana.spulber@icdapicultura.ro (R.S.); 2 Faculty of Biotechnologies, University of Agricultural Sciences and Veterinary Medicine, 011464 Bucharest, Romania

**Keywords:** *Vairimorpha* (*Nosema*), honeybee, nosemosis control strategies, eco-friendly microbial treatments, probiotic, postbiotics, synbiotic

## Abstract

Nosemosis is an intestinal infection caused by intracellular fungal organisms from the *Vairimorpha* (formerly *Nosema*) group, which seriously harms honeybee colonies and is a factor in their worldwide decline. With the ban on fumagillin use in European apiculture and the limitations of conventional treatments, it is essential to identify sustainable alternative solutions. This study presents new environmentally friendly microbe-based strategies to prevent and treat infection, focusing on probiotics, postbiotics, synbiotics, and mixes with plant extracts, as well as suggesting a new approach for the future. This review discusses the latest results based on using beneficial bacteria (e.g., *Lactobacillus* and *Enterococcus faecium*) and their byproducts to decrease the spore levels and modulate the gut bacteria pattern. Moreover, innovative approaches, such as genetically engineered gut bacteria to target pathogen gene expression through RNA interference, have been mentioned. Although results vary depending on microbial strain, delivery method, season, and ecological context, microbial treatments represent a promising, safe, and adaptable alternative for modern apiculture. The paper is necessary to validate these strategies’ real-world efficacy and to develop standardized microbial formulations suitable for practical implementation by beekeepers.

## 1. Introduction

In recent years, honeybees—one of the highly efficient plant pollinators—have been declining due to habitat loss, climate change, pesticide exposure, and diseases produced by microbial pathogens, whose virulence can be increased by environmental stressors [1]. A recent large-scale standardized survey proved that bee populations are facing worldwide escalating colony losses, particularly in Europe and the United States where on average 30.4% of honeybee colonies and 39.6% of stingless bee colonies are lost per year [2]. The increase in colony losses of managed honeybees is partially attributed to the parasitic and pathogenic attacks associated with exposure to pesticides and nutritional stress which make honeybees more susceptible to diseases [3]. Among honeybee diseases, nosemosis is one of the most prevalent.

Nosemosis disease in honeybees is caused by intracellular spore-forming microsporidian fungal parasites of the *Vairimorpha* (formerly *Nosema*) genus, with three species found—*V. apis*, *V. ceranae*, and *V. neumanni*—that differ in virulence, geographical range, and clinical signs of infection [4,5,6]. Research indicates that *V. ceranae* is more virulent than *V. apis*, while *V. neumanni* found in Uganda does not appear to be a virulent strain for Ugandan bees [3]. *V. apis* primarily infects European/western honeybees (*Apis mellifera*), while the more prevalent and emerging pathogen *V. ceranae* has a broader host range among honeybees and has also been found in Asian (*Apis cerana*) honeybees and bumblebees (*Bombus* spp.). *Vairimorpha* prevalence varies significantly by geographic region, climate, and the specific honeybee population, with infection rates of over 50% in some regions [1,3].

Nosemosis is a disease that damages bee health by disrupting digestion, reducing immune function, and leading to colony decline. The life cycle of *Vairimorpha* (*Nosema*) species, mainly *V. apis* and *V. ceranae*, are similar. The fungal microsporidian parasites produce spores which are ingested by the adult honeybees when they feed on contaminated food and water, from contaminated comb materials or hives. After ingestion, fungal spores can germinate in the ventriculus, invading the epithelial cells of the bee midgut. The fungal parasite multiplies within the gut cells, ultimately producing more spores which can be eliminated in feces and then infect other bees [3]. This infection weakens the insects, as it can cause gut lesions, suppress cellular defenses, and reduce vitellogenin expression, an important protein for bee health and longevity [7]. Finally, it leads to chronic infections, reduced lifespan of worker bees, impaired foraging efficiency, and increased colony losses. Nosemosis is a disease that specifically affects the honeybee’s intestinal tract, which harbors a stable community of microbes that play essential roles in digestion, immune function, and disease resistance. Thus, it is supposed that infection with *Vairimorpha* (*Nosema*) spp. imbalances honeybee gut microbiota which will contribute to weakening the bee’s health.

The gut microbiome, which is the most studied part of the honeybee microbiota, consists of synbiotic bacteria and fungi, a microbial community found mainly in the honeybee ileum and rectum [8]. The worker honeybee gut contains core bacterial species including *Snodgrassella alvi*, *Gilliamella apicola*, *Lactobacillus* Firm-4 and Firm-5, and *Bifidobacterium asteroides* [8]. In addition to their role in bee nutrition, core and some other bacteria have been proven to help the *A. mellifera* immune system fight and protect against pathogen infection. Thus, the core bacteria *S. alvi* and *G. apicola* have been suggested to produce protective biofilm against pathogen infection, while the non-core *Bombella apis* protects hives from fungal infection [9,10]. Only 1% of the microbiome is suggested to be composed of yeast and fungi, while yeasts such as *Wickerhamomyces anomalus* are considered a general indicator of honeybee stress [11,12]. It has been shown that environmental and biotic stressors such as pesticide exposure, nutritionally poor-quality diet, and pathogen infections can disrupt the bee microbiome (dysbiosis), leading to weakened immune systems and increased mortality rates [1,13]. Some relevant studies have proven the association between infection with *Vairimorpha* (*Nosema*) spp. and dysbiotic microbiota, but the mechanisms underlying this association were less investigated [12,14,15,16,17,18]. Moreover, researchers have demonstrated that colony-level variation in *Vairimorpha* (*Nosema*) spp. susceptibility cannot be explained only by host genetics and suggested that the insect microbiome might contribute to this variability [19]. Furthermore, research has shown that the intensity of infection was associated with specific bacterial and fungal strains from honeybee gut microbiota that encouraged using microbial-based treatments for nosemosis prevention and treatment [11,12,18]. Also, nutritional stress that leads to a reduction in pollen availability and diversity has been proven to impact the honeybee microbiota with subsequent consequences on honeybee immunity while favoring the multiplication of *N. ceranae* [1].

The most common therapy used in the past to fight nosemosis is the terpenoid fumagillin, originally isolated from the fungus *Aspergillus fumigatus*. However, its use has been forbidden in Europe since 2012. Numerous studies have proven that mycotoxins are absorbed mainly by the midgut, which is the main site of fungal infection, but these substances could lead to severe gut dysbiosis based on their antimicrobial activity [20,21,22]. Additionally, concerns have been raised about residue risks in honeybee products and the negative effects of fumagillin [21,22]. Therefore, there is a need for innovative, effective, and eco-friendly treatments for controlling *Vairimorpha* (*Nosema*) spp. based on natural products, such as plant extracts or essential oils and microbial-based products to prevent and/or treat nosemosis [13,23,24,25,26].

Given the alarming decline in honeybee populations and the consequential threat to the apiculture industry, agricultural productivity, and biodiversity, this study provides valuable insights into natural microbial-based treatments aimed at supporting honeybee populations. Several studies list in detail knowledge about natural substances, synthetic agents, and probiotics used to treat honeybees, including alternatives that fight nosemosis [13,27,28]. This review builds upon the previous information in order to discuss promising eco-friendly microbial-based strategies to control nosemosis while highlighting recent developments in the field and suggesting new approaches to design further experiments. Sustainable beekeeping focuses on bee-friendly solutions that protect honeybees from different threats (e.g., diseases, climate change, pesticides), while also considering the impact of apiculture on the environment. Implementing eco-friendly beekeeping to maintain healthy pollinator colonies includes using field-relevant doses of natural treatments to protect and treat honeybee diseases, as well as practices that encourage the bees’ own resilience and natural defenses.

## 2. Nosemosis Control Through Eco-Friendly Microbial-Based Treatments

In the case of nosemosis prevention and control, we focus on maintaining strong, healthy hives based on good beekeeping practices and targeted treatments in case of disease [29]. Traditionally, nosemosis has been managed using antimicrobial agents (fumagillin, fumagillin-B-dicyclohexylammonium salt); however, concerns regarding antibiotic resistance, chemical residues in hive products, and environmental impacts have led to the exploration of alternative treatments. The antibiotic reduction in beekeeping has prompted ongoing exploration of the natural alternatives for treating nosemosis, including using plant bioactive molecules and beneficial microorganisms that have shown effectiveness in reducing fungal pathogen spores and improving bee colonies’ survival [13,27].

The first alternative treatments to fight nosemosis were based on various preparations made entirely of herbal ingredients with antiparasitic action on pathogens but without taking into consideration the effect on the bee intestinal microbiota. Different plant extracts and essential oils were selected as natural solutions based on their content of biologically active compounds that can reduce the level of pathogens, increase the bee’s immune system, and prolong the honeybee’s lifespan [13,24,26,30,31,32,33]. All studies aimed to identify the most effective treatment against the two species of *Vairimorpha* (*Nosema*) spp., *V. apis* and *V. ceranae*. Thus, thymol, an extract from *Thymus vulgaris*, proved to have both antifungal and antiparasitic properties in bees and is known for its ability to suppress another honeybee pathogen, *Varroa destructor*. Thymol’s effectiveness as a treatment option comes from its low toxicity and low residuality within honey [30]. In vitro experiments have proven that thyme oil and its constituents inhibit the growth of several fungal and bacterial honeybee pathogens [31,32,33,34]. A reduction in the development of *V. ceranae* after administration of thymol solution alone or the combination of thymol–resveratrol or thymol–propolis has been reported by different studies [35,36,37]. Also, propolis has a phytochemical composition that depends on the local flora but is rich in flavonoids, phenolics, esters, and terpenes, and it has been demonstrated to reduce the level of *Vairimorpha* (*Nosema*) spp. [26,38,39,40,41]. Moreover, thymol and propolis extract applied to *Nosema*-infected bees decreased the genotoxicity effect and increased the expression of bee antimicrobial genes while decreasing the activities of oxidative enzymes [35]. In parallel, there is strong support for the crucial role of propolis, which can stabilize the honeybee microbiome, but more research is required to understand how propolis ingestion might influence the bee gut microbiota [42,43,44]. Similarly, research has shown that some commercial mixtures of plant extracts significantly influence the biocontrol of nosemosis, such as two phytotherapeutic products, Protofil and Nozevit, containing mixtures of essential oils and polyphenols extracted from plants [45]. However, for some natural plant products on the market that claim to protect honeybees from mortality caused by *Vairimorpha* spp., the outcomes are contradictory. Nozevit is a blend of plant polyphenols, Honey-B-Healthy^®^ is composed of lemongrass and spearmint essential oils, and HiveAlive^®^ is a bee health supplement containing a blend of seaweeds, thymol, and lemongrass. All of these products claim that the treatments improve bee nutrition and colony survival after *Vairimorpha* (*Nosema*) spp. infection [23,25]. However, a recent study challenged the efficacy of these three commercial products currently used by beekeepers to control microsporidian pathogens [5]. The authors concluded after lab and in-field experiments that the fungal parasite decrease is more likely related to the phenology of spore prevalence and intensity in honeybee colonies than to phytochemical treatments. However, Ewert et al., 2023, have demonstrated in field experiments that lemongrass and spearmint essential oils, as well as the propolis extracts, increased the bees’ lifespan and benefited their health, while the abundances of core gut microbiota taxa were largely unaffected by host consumption of the tested plant extracts [44].

As accumulating evidence has suggested the importance of gut microbiota in honeybee health, few studies have tested the combined effect of plant extracts and selected bacterial strains in controlling natural infection with the fungal parasite *Vairimorpha* (*Nosema*) spp. Thus, Garrido et al. (2024) investigated under laboratory and semi-field (cage tests) conditions the administration of the plant-based product HiveAlive^®^ and selected bacterial strains isolated from honeybee microbiota (*Bifidobacterium coryneforme* and *Apilactobacillus kunkeei*) on *V. ceranae* infection [46]. They proved a significant and rapid reduction in pathogen load after bacterial mixture and plant extract treatment, regardless of the composition of the diet, but this bioactivity was seasonally linked. The authors noted that *A. kunkeei* (former *L. kunkeei*) was even more effective than the bacterial mixture, a fact that can be correlated with the ability to restore the gut symbiotic communities in case of dysbiosis and decrease the counts of *V. ceranae* spores [47,48]. The researchers concluded that bacterial strains’ selection, as well as the time and frequency of the administration, strongly influence the results of the treatments [46,47,48]. Additionally, Alberoni et al. (2021) have shown that a microbial-based strategy has a positive effect if it does not produce far-reaching microbiome changes in the gut microbiota of the host that might induce dysbiosis [49]. The effect of the administration of HiveAlive^®^ with thymol and a mixture of beneficial bacteria (*Lactobacillaceae* and *Bifidobacterium* spp.) on the gut microbiota of honeybees and observed significant variations in the modulation of bee gut microbiota composition was analyzed. Microbial detection using both real-time PCR and next-generation sequencing has revealed that administered strains were detected in the gut, while the bacterial blend produced only minor changes in the microbiome profiles, especially in the population of *Snodgrassella* spp. On the other hand, the plant mixture increased the abundance of *Bartonella* spp., which can use plant secondary metabolites and has a beneficial effect on honeybee health. A reduction in the *Lactobacillus* spp. Firm-4 (reclassified to *Bombilactobacillus* spp.) population was recorded after both plant and microbial treatments. Overall, the study highlighted the importance of bio-based approaches “with respect to the honeybee microbiota composition” [49].

Therefore, nosemosis control based on bee microbiota modulation has gained attention for its potential to enhance honeybee health, as well as to combat infections naturally and sustainably. Probiotics and synbiotics have been explored as a potential treatment for nosemosis, with a focus on the effects of oral supplementation, particularly with lactic acid bacteria [27,50]. The microbial strategy is based on the probiotic microorganisms that contribute to gut homeostasis by outcompeting pathogenic microbes and preventing *Nosema* spores from attaching and proliferating, improving nutrient absorption and metabolism, and enhancing host immune responses. In honeybees, probiotics mainly consist of beneficial bacteria from the genera *Lactobacillus*, *Bifidobacterium*, and *Enterococcus*, gut microbial symbionts, associated with the intestinal system of vertebrates and invertebrates [27]. They can play an important role in the prevention and treatment of microflora dysbiosis, and several studies have emphasized that they can help to control bee parasites [50,51,52,53,54,55]. However, Zhang et al. (2021) debated that increasing indigenous intestinal microbiota (especially lactobacilli and bifidobacteria) might increase the bee population, but it does not help in microsporidian fungal control [15]. The hypothesis is supported by the positive association between *V. ceranae* infection and some gut core bacteria species (e.g., *G. apicola*, *Lactobacillus* spp., *Bifidobacterium* spp.) found in a few independent studies [16,17,51]. These positive associations benefit both gut bacteria and *V. ceranae*, as the pathogen has adapted and can modulate the intestinal microbiota to maintain host homeostasis, inducing tolerance to the infection but a high prevalence of the disease. The results of the study do not clarify the mechanism of these positive associations, but consider that the interaction between pathogen and microbiota is important to sustain host survival and is beneficial for the pathogen [15]. Therefore, a new strategy based on the bee’s beneficial intestinal strain *Apilactobacillus kunkeei*, which controls fungal pathogens and gut opportunistic bacterial pathogens that are promoted by *Nosema* infection (e.g., *Serratia* sp.), has been investigated [14].

In recent years, under laboratory conditions, an increased number of studies have explored various probiotic strains and their impacts on critical parameters, including honeybee survival rate, colony strength, honey production, and general bee health or the effect on the immune response of bees to *Vairimorpha* (*Nosema*) spp. [36,51,52,53,54,55,56,57]. Field trials have further supported these findings, demonstrating that probiotic supplementation can be a viable method for managing nosemosis without the drawbacks of synthetic chemicals. The first experiment with probiotic native strains isolated from the gut of healthy honeybees to manage nosemosis was performed in 2016 by Baffoni and co-workers. They fed healthy bees and bees artificially infected with *V. ceranae* spores using sugar syrup supplemented with indigenous strains of bifidobacteria and lactobacilli strains [52]. Eight days after infection, real-time PCR results confirmed that the parasite level in probiotic-treated honeybees was significantly lower compared to results obtained with infected bees from the control group, fed with sugar syrup. However, a later work concluded that the hindgut native population of *Bifidobacterium* spp. might not prevent the effects of *Nosema* infection [51]. The authors found a significant and positive correlation between bifidobacteria abundance and *V. ceranae* infection, but the specific cause and mechanism of this association were not clarified. In another work, a mixture of four native strains of *A. kunkeei* was used as a probiotic food additive to feed infected larvae and honeybees in controlled laboratory experiments [48]. This beneficial bacterial mixture decreased the mortality associated with *Paenibacillus larvae* bacterial infection in larvae and the counts of *V. ceranae* spores from adult bees. Similarly, *L. salivarius* A3iob has been proven to reduce the incidence of both parasites *Varroa destructor* and *Vairimorpha* (*Nosema*) spp. [53]. Importantly, the spore levels of *Vairimorpha* spp. in probiotic-treated colonies depended on the apiary and the year of application, but a significant decrease was mainly observed in the post-winter period [53]. Another study investigated the effect of endogenous strain *Lactobacillus johnsonii* CRL1647 isolated from the gut tract of a worker bee on gut microbial load, honey production, *Nosema* index, and *Varroa* incidence [54]. Similarly, honey production was obtained after food supplementation of honeybees with log 5 CFU/mL lactobacteria every 15 days or 30 days. Moreover, in both cases, the *Nosema* index was reduced, while *Varroa* incidence decreased when *Lactobacillus* spp. was delivered monthly. The administration of lactobacilli every 15 days increased the total number of aerobic microorganisms and bacteria from the genera *Lactobacillus* and *Enterococcus*, while the number of enterobacteria was constant but below that of the control group at the end of the experiment. The probiotic supplementation demonstrated dampening effects on parasite load and many beneficial effects on parameters like increased honey production or queen reproduction and productivity [54]. *E. faecium* is a commensal bacteria from the honeybee midgut that produces lactic acid. This metabolite causes thickening of the peritrophic membrane in the ventriculus epithelium, which protects epithelial cells against *Nosema* infection [55,56]. Borges et al. (2020) compared Protexin^®^ (*E. faecium* single strain), Protexin Concentrate multi-strain (*Lactobacillus acidophilus*, *L. plantarum*, *L. rhamnosus*, *L. delbrueckii*, *Bifidobacterium bifidum*, *Streptococcus salivarius*, and *E. faecium*), and Vetafarm Probiotic and dietary fiber prebiotics, such as acacia gum, inulin, and fructooligosaccharides, to evaluate their effect on *V. ceranae* spore loads and honeybee colony survival [36]. The single-strain probiotic significantly decreased *Nosema* spore numbers (59%) and increased bee relative survival to both *V. ceranae* inoculated and non-inoculated bees [35]. Infected bees fed 3.75 mg/mL Vetafarm Probiotic or 2.50 mg/mL Protexin Concentrate single-strain had significantly higher survival rates than both *V. ceranae*-inoculated and non-inoculated bees [36]. A more recent example of a potential probiotic was provided by Sbaghdi et al. (2024), who successfully used the lactic acid bacterium *Pediococcus acidilactici* MA18/5M to improve honeybees’ tolerance to the fungal parasite *V. ceranae* [57]. Also, other commercial probiotics (Bactocell^®^, Levucell SB^®^ Biogen-N, Trilac, Lakcid, etc.) have been tested as an alternative therapy against *V. ceranae* infections in honeybees [49,58,59,60,61,62]. Differences between the reported results could be attributed to differences in experiment design, the doses used, the targeted gut microbial populations, and so on [62]. Accumulating evidence from some works suggested that endogenous bacterial strains could have higher or similar efficiency as commercial probiotic non-native strains in terms of the survival of honeybees infected with *V. ceranae*. However, uncontrolled and unbalanced administration of probiotics to honeybees may increase pathogen susceptibility related to dysbacteriosis [17,18,61,62].

Furthermore, researchers demonstrated that gut bacterial metabolites did not have a toxic effect on bees while proving to have antiparasitic activity on *Vairimorpha* (*Nosema*) spp. Thus, the bacterial metabolites produced by *Bacillus* and *Enterococcus* strains (surfactin and bacteriocin) isolated from the bee midgut and honey inhibited the development of *V. ceranae* [63]. A particular surfactin was proved to act either by direct exposure to parasite spores and reducing their infectivity or in the honeybee digestive tract which interferes with parasite development [62]. Also, oral administration of metabolites or cell-free supernatants from *L. johnsonii* containing mainly organic acids decreased *Nosema* prevalence [63,64]. In laboratory conditions and field trials, the oral administration of metabolites from *L. johnsonii* AJ5 showed that the product increased colony populations and enhanced bee health [63]. A higher dose of organic acids (60 uL/honeybee) such as lactic acid, phenyl-lactic acid, and acetic acid did not cause honeybee mortality and could decrease the bee’s intestinal pH, which inhibits the development of pathogenic microbes [55,65,66]. Another study has suggested that oxalic acid can be used both in the laboratory and in field conditions to control *V. ceranae* infection [67]. Some oxalic-based treatments, such as ApiHerb and Api-Bioxal reduced the abundance of *V. ceranae*, but ApiHerb also decreased the prevalence of infected bees [25]. This positive influence on the health condition of bee families suggested the idea to combine the organic acids prebiotic products (lactic acid or acetic acids) with probiotic bacterial strains (*L. acidophilus* LA-14, *Bifidobacterium lactis* BI-04, and *L. casei*) [66]. Similarly, postbiotics from beehives have gained attention for their potential to enhance bee health and combat *Varroa destructor* infections in a natural and sustainable manner [68]. In another study, cell-free supernatant obtained via culture of *L. johnsonii* has provided promising results and increased the mortality of this parasite [69]. The postbiotic product is defined as “non-viable bacterial products or metabolic products from microorganisms that have biological activity in the host” [70]. Postbiotics are generated by live bacteria and have been proved to have numerous potential health benefits for affected hosts, including gut microbiota modulation, increased antioxidant activity, and immunomodulatory effects. However, according to the present state of knowledge, there is no information about the postbiotic preparation effect on *Vairimorpha* (*Nosema*) spp. infection. In parallel, there is strong support for using postbiotics to combat bee pathogens as they have in their composition antimicrobial substances that can reduce pathogens’ development and influence. Some of them can adjust and cause the pH levels to increase, which later leads to the growth of many beneficial microorganisms. Also, they could include structural components like lectins and fimbriae, which can inhibit the growth of pathogens based on competitive adhesion, thus providing the opportunity for beneficial microbes to develop [71]. Some bacteria such as lactic acid bacteria produce metabolites, such as ethanol, acetic acid, lactic acid, butyric acid, and bacteriocins, that remain intact after processing and can be used as antimicrobials [72]. Therefore, postbiotics may serve as a valuable tool for improving the health and welfare of bees and in nosemosis management.

It has to be mentioned that several works have investigated the effectiveness of synbiotics in preventing and reducing *Nosema* infections. Synbiotics contain a combination of probiotic strains and prebiotic substances (e.g., inulin and chitosan). A study on the commercial probiotic strain of *Lactobacillus rhamnosus* that exhibited a high rate of survival in sugar syrup (56.56%) and prebiotic inulin examined their efficacy in preventing and treating nosemosis, including the impact on the survival rates of bees infected with *V. ceranae* [29]. Surprisingly, honeybees fed 9 days prior to fungal infection with sucrose syrup supplemented with the commercial probiotic showed a 25-times-higher number of microsporidian spores than the control group that was not treated with prebiotic and probiotic. Conversely, inulin supplementation alone at a concentration of 2 μg/mL did not significantly affect honeybees’ survival or the nosemosis infection level [29]. This work suggested that non-native probiotics might increase susceptibility to pathogen infection and shorten the bee lifespan. However, other studies testing naturally occurring probiotic strains have demonstrated a reduction in parasite loads and multiple benefits, such as higher honey production, improved queen reproduction, and overall colony productivity. Klassen et al. (2021) fed *A. mellifera* bees with sugar syrup or protein patties supplemented with commercial probiotic Protexin^®^ containing *E. faecium* (2 × 10^12^ CFU/kg) and prebiotics (eugenol, chitosan, and naringenin) and evaluated their effects on *V. ceranae* infection and colony survival in a field study [73]. The most promising combination was the probiotic and naringenin both for *Nosema* infection biocontrol and to increase the adult bee population. Table 1 shows a comparison between probiotic, postbiotic, and synbiotic products used for the prevention and treatment of nosemosis.

There is a long history of research about bacteria from honeybee gut microbiota, while we still know very little about other microbes, such as resident yeast, and their effect on nosemosis prevention or treatment. Over a decade ago, Ptaszyńska et al. (2016) suggested that a poorly understood yeast community from the honeybee gut was affected by *Vairimorpha* (*Nosema*) spp. infection [12]. Thus, fungal infection promoted an increase in the intestinal yeast population, leading to additional opportunistic yeast infections that increased the bee’s mortality. Progressions were performed in a recent work that evaluated the commercially available product EM^®^ PROBIOTIC FOR BEES on *Nosema* spp. infection level and colony survival [74]. Long-term treatment with the probiotic, containing multiple species of lactic acid bacteria, yeast, and photosynthetic bacteria, mitigated the effects of fungal infection on honey gut microbiota, increasing *Snodgrassella alvi* and recovering the *Bifidobacterium* population. In another work, Tauber et al. (2019) suggested that the honeybee midgut yeast *Wickerhamomyces anomalus* population decreased during nosemosis and influenced the relationship between the gut-residing bacteria, especially *Lactobacillus* Firm-5 [11]. These works emphasize the significance of maintaining a balanced gut microbial community in honeybees.

Genetically engineered honeybee gut microbial strains are considered a potential breakthrough for pollinator health, as they provide an alternative for improving honeybee health. The methods typically involve emerging techniques such as CRISPR-Cas9, plasmid introduction, and gene knock-ins or knock-outs to improve microbial function or inhibit parasite infection [75]. Core bacterial species such as *Snodgrassella alvi*, *Gilliamella apicola*, and *Lactobacillus* species contribute to honeybee health by aiding in digestion, regulating immune responses, and defending against pathogens; therefore, these species were targeted by genetic engineering [76]. First, *Snodgrassella alvi* was engineered to target double-stranded RNA against *Varroa destructor* through the bee RNA interference (RNAi) pathways [77]. All of the honeybees colonized with the engineered *S. alvi* strain showed suppressed viral proliferation. The study was conducted at the laboratory level and proved that the symbiont-mediated RNAi approach is a great tool for both studying bee functional genomics and protecting bee health. Recently, another work developed an engineered *S. alvi* strain that inhibits microsporidia proliferation using nanoparticle-mediated RNA interference [75,78,79]. The modified gut bacteria represses *V. ceranae* gene expression, delivering double-stranded RNA corresponding to genes involved in the redox system of the microsporidia, which are essential for the infection process [78]. Thus, engineered microbes that interfere with pathogen colonization or can produce antimicrobial substances could reduce infection rates and improve bee survival. Genetically engineered bee gut strains represent a promising avenue for enhancing pollinator health and mitigating the challenges facing worldwide honeybee populations. However, safety issues related to unintended ecological consequences, regulatory oversight, and ethical considerations must be addressed in further research before widespread implementation of this approach.

**Figure 1:**
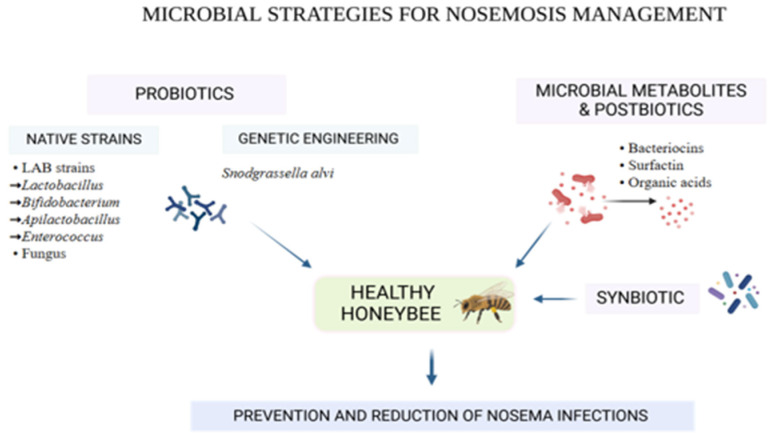
Microbiota-targeted strategies for nosemosis management and control.

A graphical summary of the main microbiota-targeted strategies for nosemosis management and biocontrol is provided in Figure 1.

Microbial-based solutions present a promising, eco-friendly alternative to traditional chemical treatments for nosemosis in honeybees. By enhancing specific gut microbial strains that boost immunity and reduce *Nosema* proliferation, these products contribute to healthier colonies and more sustainable beekeeping practices. Continued research and practical implementation of microbial-based strategies will be essential in ensuring the long-term viability of honeybee populations and the pollination services they provide.

## 3. Microbial Supplements for Nosemosis Control: Formulation, Delivery Methods, and Implementation Strategies

Maintaining worldwide healthy and productive populations of honeybees (genus *Apis*) is essential for sustainable agriculture and preserving biodiversity. As concerns about colony collapse disorder, pesticide exposure, and pathogen prevalence intensify, interest in microbial supplements, particularly probiotics and synbiotics, has grown. The potential benefits of microbial supplementation include improved and stabilized gut microbiome, as strengthening the beneficial microbes in the bee’s gut improves nutrient absorption and metabolization, as well as resistance to environmental and pathogenic stressors, and enhanced immunity—factors that subsequently lead to the increased viability and stability of bee colonies. However, their direct incorporation into the bee diet brings challenges due to stability, solubility, and bioavailability issues. Thus, formulation science is critical in effectively integrating these microbial products into supplements or other delivery systems for honeybees.

A probiotic formulation can be defined as one strain or a specific combination of microbial strains, mainly bacteria or yeast, designed to have a beneficial effect on the host’s health. Probiotic and synbiotic formulations can be used as honeybee dietary supplements integrated into their diet to promote overall hive health, to stave off infectious disease, or for treatments, as these products can inhibit different bacterial and fungal pathogens and significantly reduce levels of toxic byproducts or antibiotics [12,18,36,73]. These microbial products are often used to colonize or repopulate the bee’s gut with beneficial microbes. The honeybee gut microbiota contains a unique and relatively simple microbial community, dominated by genera such as *Lactobacillus*, *Bifidobacterium*, *Gilliamella*, *Commensalibacter*, *Bartonella*, *Snodgrassella*, and *Frischella* [79]. Knowledge in this subject is progressing and, in recent years, there is more research comparing the normal honeybee gut microbiome with microbiota from *Nosema*-infected bees. In the infected bees, there were higher amounts of fungi, including yeast, and bacterial groups such as Firmicutes (e.g., Lactobacillus), γ-proteobacteria, Neisseriaceae, *Serratia*, and other unidentified bacteria, while in healthier bees there were bacteria from groups like Orbales, *Gilliamella*, *Snodgrassella*, and Enterobacteriaceae [12,14,16]. However, some reports were contradictory; thus, Tlak Gajder et al., 2023, showed that the relative proportion of *Bifidobacterium* spp. was lower after *Nosema* infection, while Zhang et al. (2019) suggested that bifidobacteria abundances were significantly and positively associated with *V. ceranae* infection [51,74].

Although published analyses of probiotic strain persistence in the honeybee gut are scarce, numerous studies have proved that non-native probiotics do not persist for a long time in the host, can disrupt the delicate microbial balance, and produce limited functional outcomes [27]. Thus, Anderson et al. (2024) have demonstrated that commercial probiotics with non-bee gut origin strains do not help bees with the negative effects of antibiotic-induced gut dysbiosis [80]. In the case of studies on *Nosema*-infected adult honeybees, the results are contradictory but most of the experiments proved that non-native bacterial strains are transient, as well as their effects on fungal spore counts and the survival rates of honeybees [27]. Therefore, to be efficient, the microbial supplements must be formulated using specific strains that are either native to or well-tolerated by the honeybee gut [27,49]. Moreover, microbial formulations at the hive scale should have a different range of probiotic strains compared to those used for adult honeybees. Some major commercial microbial products available on the market contain a wide range of beneficial probiotic strains, such as *Lactobacillus* spp., *Apilactobacillus* spp. (*L. kunkeei*), *Lactiplantibacillus* spp. (*L. plantarum*), *Enterococcus faecium*, *Bifidobacterium* spp., *Bacillus* spp., the yeast *Saccharomyces cerevisiae*, and the fungus *Trichoderma reesei*.

Studies have shown that honeybees can benefit from microbial supplementation within a range of concentrations, although no consensus exists about the minimal concentration to be used in probiotic formulations. The optimal concentration depends on the specific probiotic strain and delivery method, while most manufacturers recommend 10^5^–10^8^ CFU/mL. In the case of sugar syrup supplemented with the selected probiotic strains, these microbial cells should exhibit a high rate of survival in these conditions to reach the minimum concentration that induces the probiotic effect [18]. Another important aspect is that the microbial products must be formulated considering the strain tolerance to environmental conditions like pH, temperature, and humidity. Stabilizers are used to preserve microbial viability during storage and application. Furthermore, microbial supplements must be free from contaminants, antibiotic-resistant genes, or unintended metabolic byproducts. Ensuring the safety of microbial supplements for honeybees is important but regulatory frameworks for microbial additives in apiculture are still emerging in many countries.

Effective administration of microbial supplements to honeybees requires delivery systems that ensure microbial viability and targeted colonization. Microbial formulations can be delivered directly as supplements or via feeding through sugar syrup, pollen patties, or other formulations used for honeybee feeding. The common delivery methods are summarized in Table 2. A recent study suggested that honeybees feeding with a sugar-based diet were more vulnerable to *Nosema* infection; thus, incorporation of probiotic strain into the sugar syrup might affect their efficiency [16]. Administration schedules are important for bee colony performance and less important for the *Nosema* infection index; thus, honeybees treated once and twice a month with the native strain *L. johnsonii* CRL 1647 exhibited a reduction in the number of total spores at the end of the treatment [54]. Another work has proved that bacterial strain selection, as well as the time and frequency of the administration, strongly influence the efficacy of treatments used to control natural infection of *V. ceranae* in honeybee colonies [46]. Moreover, the seasonal pattern of parasite fungal infection is important for the efficacy of microbial treatments [53]. The viability of strains from microbial products can be compromised by different factors, such as storage conditions, temperature, and UV exposure, the delivery method, and hive temperature fluctuations, requiring robust encapsulation or stabilizing technologies. Few studies evaluated the effects of delivery systems to protect the encapsulated microbial products through polymer enhancements and coatings, a process that is influenced by a challenging physiological regime [28,29].

Moreover, the compatibility of microbial supplements with other hive interventions, including acaricides, and antibiotics, must be carefully assessed; as some treatments may inhibit microbial survival or interfere with the gut re-colonization. On the other hand, environmental conditions such as climate, local flora, pesticide exposure, and disease prevalence significantly influence the efficacy of microbial supplements. The combination of various stressors (e.g., climate changes, pesticides, poor nutrition) working together with the *Nosema* infection creates a greater negative impact on the honeybee colonies than any single factor alone. Therefore, microbial supplementation strategies should be tailored to specific regional and seasonal contexts. The meaningful observations from in-cage or small in-field studies, which indicate the beneficial effect of microbial products on *Nosema* infection control, should be strengthened by in-field trials under diverse environmental conditions, that are essential to validate the real-world performance of these supplements.

The practical implementation of microbial products to control nosemosis requires economic feasibility and proven efficacy. These microbial supplements offer promising benefits, but their widespread adoption depends on cost, ease of use, and demonstrable return on investment for beekeepers. The implementation of strategies to keep nosemosis under control begins with biosecurity measures, including good beekeeping practices (GBPs) that provide essential solutions to avoid the spread of disease among the colony and other different apiaries [81]. Furthermore, beekeepers incorporating microbial products for nosemosis management should ensure consistent application, especially during stressful periods, such as seasonal transitions or hive transportation. Additionally, maintaining a diverse and natural diet for bees supports the proliferation of beneficial microbes from honeybee gut microbiota that fight pathogens.

## 4. Challenges and Future Directions of Naturally Microbial Treatments for Nosemosis Control in Honeybees

Ongoing research into the bee microbiome and the effectiveness of microbial supplements is crucial for developing reliable microbial products for nosemosis prevention and treatment. Despite promising results, several challenges remain in the widespread adoption of microbial-based treatments for nosemosis control which are summarized in Table 3. The provocations include the need for strain-specific research, to identify and test new beneficial microbes for inclusion in microbial-based products, standardization of probiotic formulations, and developing more targeted delivery systems that enhance the effectiveness of supplements [8,13,27,50]. Also, it is important to determine which are the most suitable methods that should be combined and to find the right way and moment to adopt them to reach their maximum potential. Furthermore, the practical implementation of microbial supplementation in beekeeping is plagued with biological, logistical, and regulatory challenges.

While challenges remain in finding effective and sustainable alternative treatments for nosemosis, the key will be to develop solutions that are both effective against the disease and friendly to bees and the environment (Table 4). More research is needed to understand the mechanisms involved and the consequences of *Nosema*-induced dysbiosis, which will help to evaluate the potential of microbial-targeted strategies to mitigate these challenges [8,13]. Moreover, future studies should focus on investigating the long-term effects of microbial supplementation on bee health and productivity and understanding their interactions with honeybee gut microbiota, while assessing their effectiveness under varying environmental conditions.

Ideally, microbial supplementation to fight nosemosis should be integrated into holistic hive health management plans that minimize chemical dependence and leverage natural immunity and microbial ecology.

## 5. Conclusions

The exploration of natural microbial treatments for nosemosis control offers valuable solutions at a time when honeybee colony health is under severe threat. Accumulated evidence highlights the effectiveness of probiotics and synbiotics in restoring gut microbiome balance, reducing spore load, and improving colony parameters such as survival, honey production, and reproductive activity. Moreover, microbial metabolites and postbiotic preparations may serve as valuable treatments for improving the health and welfare of bees and in nosemosis management. However, the practical implementation of microbial-based treatments to fight *Vairimorpha* (*Nosema*) spp. requires careful attention to microbial strain selection, delivery methods, environmental compatibility, safety, and beekeeper engagement. Using these products in routine hive care means translating the laboratory insights into effective, field-ready tools for modern apiculture. Thus, these tools could reshape beekeeping into a sustainable industry.

## Figures and Tables

**Table 1:** Probiotics, postbiotics, and synbiotics—microbial sources, role, and benefits for honeybee health.

Classification	Definition	Role	Benefits	Microbial Sources
Probiotics	Live microorganisms with a large scale of benefits	Improve gut microbiota composition, increase immunity, enhance gut barrier function, produce antimicrobial substances	Restoring gut microbiome balance, improving the absorption of nutrients, reducing spore load, improving colony parameters (e.g., survival, honey production, and reproductive activity)	*Lactobacillus*, *Apilactobacillus Bifidobacterium*, *Enterococcus*, *Pediococcus*, *Streptococcus*
Metabolic byproducts and Postbiotics	Metabolic byproducts of probiotic bacteria and non-viable microbes	Provide antioxidant effects, modulate gut health, improve immune response		*Lactobacillus*, *Bifidobacterium*, *Bacillus*
Synbiotics	The combination of probiotics and prebiotics	Improve gut health, increase and support the survival of probiotics		Supplements, functional feeds with synergistic effects

**Table 2:** Several methods that are being explored for delivery of the microbial supplements.

Feeding through Sugar Syrup	One of the most common methods is mixing microbial supplements into sugar syrup. This allows bees to ingest the supplements directly.
Incorporation into Pollen Patties	Since pollen is a primary food source for bees, adding microbial supplements to pollen patties can be an effective way to introduce probiotics into the hive.
Dusting with Microbial Powders	Powdered microbial supplements are dusted on the bees or hive surfaces. Bees that meet the powder ingest or interact with it.
Topical Application	Microbial preparations can be applied directly to bee exoskeleton or hive surfaces using sprays, allowing the microorganisms to interact with the bees.
Gutter or Hive Feeder Systems	Another method involves adding supplements to the hive feeder systems that distribute food throughout the colony. Systems ensure that a large portion of bees consume the supplements.
Drone or Queen Supplementation	Specific applications can be targeted at drones or the queen, as they play important roles in colony health and reproduction.

**Table 3:** The challenges of microbial treatments for nosemosis research.

Lack of Understanding of Mechanisms	While alternative treatments like herbal remedies, probiotics, and synbiotics show promise, their mechanisms of action are not always well-understood.
Efficacy and Consistency	Many alternative treatments show varying results, often depending on the dosage, timing, or environmental conditions. The natural variability in the composition of herbal or probiotic treatments can lead to inconsistent results.
Potential Harm to Bees or Hive Health	Some alternative treatments could have negative effects on the health of honeybees or the hive environment. Non-native probiotics do not persist for a long time in the host and could disrupt the delicate microbial balance and produce limited functional outcomes.
Regulatory Barriers	The regulatory landscape for alternative treatments in apiculture is complex. Many treatments that show good potential are not approved.
Resistance Development	There is concern that the overuse of certain treatments could lead to resistance, making the parasites more difficult to control.

**Table 4:** The further directions of microbial treatments for nosemosis research.

Microbiome Research	The gut microbiome of bees plays an important role in their health and immune response. By exploring ways to balance or enhance the bee microbiome, researchers will need to develop more sustainable treatments that could reduce the impact of *Nosema* infection. Probiotics, postbiotics, and symbiotic treatments could be the right alternative.
Herbal and Phytotherapy Advances	Herb and plant extracts can be used for combating *Nosema*. Flavonoids, alkaloids, and essential oils may act as natural remedies.
Genetic Approaches	By breeding or genetically modifying bees to be more resistant to *Nosema* infections, researchers are exploring natural resistance mechanisms in certain bee strains that could be selectively bred to enhance colony health.
Integrated Pest Management	The future of nosemosis treatment will likely focus on an integrated approach that combines alternative treatments with improved management practices.
Synthetic Biology	Emerging technologies like synthetic biology may enable the creation of custom solutions, such as engineered probiotics, that target *Nosema* directly.

## Data Availability

No new data were created or analyzed in this study. Data sharing is not applicable to this article.

## Abbreviations

The following abbreviations are used in this manuscript:

PCR	Polymerase Chain Reaction
CFU	Colony-Forming Units
GBPs	Good Beekeeping Practices
